# Medical Authorities, &c.

**Published:** 1884-05

**Authors:** 


### CONCEDED BY MEDICAL AUTHORITIES.

THE change from winter to spring is fraught with danger to the sick or debilitated; while even the strongest and most robust constitutions are liable to suffer from lassitude and partial failure of the nervous energies at this time. As the surface of the earth becomes more and more softened by the increased power of the sun's rays, miasmatic gases are evolved and set free, becoming the factors of malarial disorders which attack those who, in consequence of enfeebled constitutions or undue exposure, are particularly susceptible to these pernicious influences. A remedy is required, which shall not only stimulate the flagging powers of nature, but shall at the same time afford a certain degree of nourishment.

Baron Von Liebig, the famous chemist, devoted many years of his life to the production and perfection of various extracts and combinations, of which fresh beef formed the base; and at the present time combinations founded on his observations and researches are many and varied; yet nearly all these preparations of beef, whether in solid or liquid form, which are now so extensively advertised, act simply as nutritives, without containing any medicinal properties whatever — a want, the full importance of which was recognized and remedied by T. Colden, M.D., an enthusiastic advocate of Liebig's methods; the result of his observations and experience being the production of Colden's Liquid Beef Tonic, the base of which is an extract of beef, prepared after Liebig's methods but combining other important ingriedients which render it at once a nutritive, tonic, stimulant and alterative.

Thus, in cases of debility, general depression, convalescence from wasting diseases, and from all affections in which the vital powers have become weakened, together with malarial disorders generally, it is clearly indicated as an unsurpassed invigorator and builder of vital force, and its success as such has been attested by the most eminent physicians. It is no quack remedy, but its influence is sovereign and therefore needs no fulsome praise.

